# EnhanceFitness: A 20-Year Dissemination History

**DOI:** 10.3389/fpubh.2014.00270

**Published:** 2015-04-27

**Authors:** Susan J. Snyder, Meghan Thompson, Paige Denison

**Affiliations:** ^1^Partners in Care Foundation, San Fernando, CA, USA

**Keywords:** EnhanceFitness, evidence-based, dissemination, older adults, healthcare providers

EnhanceFitness (EF) is a prime example of an evidence-based physical activity program that has been disseminated far beyond its original study site. This commentary will provide an overview of the evidence, history, successes, challenges, and vision for increasing the availability and accessibility of EF. This overview is intended to be an example for evidence-based programs that want to move beyond their study sites to wider dissemination.

In 1994, researchers at the University of Washington Health Promotion Research Center (UW HPRC) and Group Health Cooperative (a health maintenance organization) collaborated with Senior Services, a non-profit community-based organization, to conduct a trial of a multicomponent disability prevention program. One hundred older adults were recruited for a 6-month study at a State of Washington senior center. Even before the pilot study ended, participants requested to keep the exercise class component of the intervention as a permanent activity at the center. Not only were the center members excited about participating, but study measures showed that the intervention group significantly improved in fitness and health: 13% improvement in social function; 52% improvement in depression; and 35% improvement in physical functioning. The control group (center members who did not participate in the program but who attended other senior center activities) deteriorated in these measures over the same period (1).

After completion of the original study, wishing to see that the successful program move beyond the original study site, partner agencies agreed that Senior Services was best positioned to hold the license for the program and oversee its dissemination. Senior Services’ dissemination strategy has been to license, train, and support community-based delivery sites that adopt EF. This strategy has been quite successful over the years and has balanced the need to maintain fidelity to the program’s protocols with the mission to expand the program’s reach in a sustainable way (2).

Since the years following the original study, from 1999 to mid-2014, EF has been offered in 34 states, at nearly 700 locations. Early on, expansion was largely due to Senior Services’ marketing of the program at the annual conference of the National Council on Aging and American Society of Aging. In 2006, the US Health and Human Services’ Administration on Aging (AoA) included EF as one of the approved programs for the Choices for Independence grants, placing it in the AoA’s highest tier of evidence-based programs (3). As a result, program adoption increased significantly in the grantee states. This growth continued in the following year (2007), when the Centers for Disease Control and Prevention Arthritis Program (CDC-AP) reviewed and classified EF as “arthritis-friendly” and it was adopted as a recommended intervention by the Arthritis Program (4). As of mid-2014, according to data collected and maintained by Senior Services from program implementation sites since 1999, EF has served over 45,000 unduplicated participants.

Throughout the program’s history, crucial support from several directions has spurred and sustained its growth. National policymakers and funders have embraced the program, prompting significant uptake far beyond the borders of the program’s home region of western Washington. In addition, these agencies have helped to fund Senior Services’ development of a comprehensive online data management platform that allows both local and centralized reporting about participant- and site-level participation and outcomes. The same platform supports Senior Services’ tracking of program licensing and funding, as well as site, staff, and training information. This internal data management infrastructure has been invaluable in allowing Senior Services to monitor program reach and fidelity, while the user-facing element allows EF’s licensees to provide meaningful reports both to their funders and to their participants, and to monitor the success of their own implementation efforts. At the other end of the spectrum from federal agencies, program participants themselves, not to mention their physicians, bolstered by subjective (5) and objectively demonstrable (6) changes in health and fitness, have been important champions for driving demand for program expansion at the community level.

EnhanceFitness has benefited from a strong continuing relationship with its original academic and healthcare research partners. This relationship has provided many opportunities to participate in research and evaluation efforts beyond the initial study. This work and the resulting articles published in professional journals (7) have ensured that the program’s protocol is kept up-to-date with the latest research in older adult fitness. Evaluation of the program’s adaptation for various cultural groups has demonstrated its ability to achieve acceptance and positive outcomes in new settings (8). Increased program dissemination to a variety of sites and populations brings increased organizational complexity as Senior Services seeks to support EF licensees in their implementation and sustainability challenges. The technicalities of managing what may be multiple new class sites, as well as recruiting, training, and retaining certified fitness professionals, are often difficult for EF licensees during implementation of the program. Most EF licensees are non-profits and sustainability in funding is a common challenge as they must pay for the initial EF license, annual license renewal fees, and instructors’ wages. The expertise of EF partners and researchers has assisted in studying solutions to these challenges.

EnhanceFitness has a vision for the future based on learning from additional research, partnerships, and funding since its original study. A new partnership with the American Council on Exercise, experts in the field of physical activity curriculum development and training, will ensure that EF instructors have the continuing education that they need to serve older adults with coexisting chronic conditions. Online delivery of training modules for instructors will help bridge the accessibility gap in remote areas for continuing education and support of that infrastructure. The program’s participation in Centers for Medicaid and Medicare Services (CMS) study beginning in 2014 is another step toward a long-time vision of EF becoming a Medicare reimbursable benefit. Continued work with the University of Washington on the adaptation of the EF program for participants with cognitive impairment will fill an ever-growing need. Expansion of locations through national partners, such as the Y of the USA and its 2,600 branches, will strengthen the EF network as a whole. Lastly, focus on increased referrals of patients from healthcare providers will fortify the links between the healthcare system and older adult wellness and self-efficacy. This is all possible based on the strong foundation built over the last two decades.

## Conflict of Interest Statement

The authors declare that the research was conducted in the absence of any commercial or financial relationships that could be construed as a potential conflict of interest.

This paper is included in the Research Topic, “Evidence-Based Programming for Older Adults.” This Research Topic received partial funding from multiple government and private organizations/agencies; however, the views, findings, and conclusions in these articles are those of the authors and do not necessarily represent the official position of these organizations/agencies. All papers published in the Research Topic received peer review from members of the Frontiers in Public Health (Public Health Education and Promotion section) panel of Review Editors. Because this Research Topic represents work closely associated with a nationwide evidence-based movement in the US, many of the authors and/or Review Editors may have worked together previously in some fashion. Review Editors were purposively selected based on their expertise with evaluation and/or evidence-based programming for older adults. Review Editors were independent of named authors on any given article published in this volume.
